# Public Health Posters Take Aim against Bloodthirsty Ann

**DOI:** 10.3201/eid2702.AC2702

**Published:** 2021-02

**Authors:** Byron Breedlove

**Affiliations:** Centers for Disease Control and Prevention, Atlanta, Georgia, USA

**Keywords:** art science connection, emerging infectious diseases, art and medicine, about the cover, public health posters take aim against bloodthirsty Ann, This is Ann–: she drinks blood!, Theodore Geisel, Munro Leaf, public health, malaria, vector-borne infections, antimalarial drugs, quinine, quinacrine, atabrine, Anopheles, mosquitoes, United States Army, World War II

**Figure:**
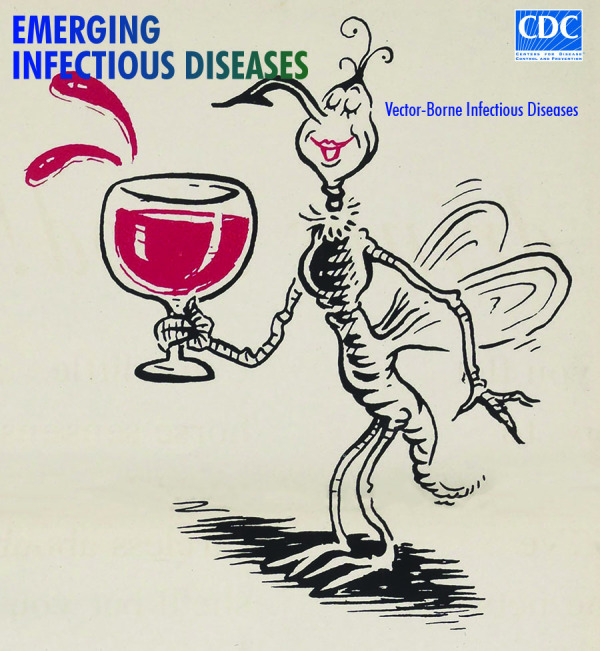
**Ted Geisel (aka Dr. Seuss) illustration; Munro Leaf, text; United States War Department Special Services Division and Government Printing Office. Detail from *This is Ann–: she drinks blood!* (1943).** Photomechanical print (poster): 35 inches x 47 inches/89 cm x 120 cm. National Library of Medicine, Bethesda, Maryland, United States. Public domain image.

From June 1942 until September 1945, the United States Office of War used various media, including posters, as communication tools. Those posters―plastered in public areas, storefronts, factories, and military installations―employed motivation, guilt, and humor to boost morale and to encourage information security, buying war bonds, planting victory gardens, and, notably for military personnel deployed to tropical and subtropical areas during World War II, preventing malaria.

For military personnel deployed to tropical and subtropical areas during WWII, the number one health problem was malaria. Because of its lingering, debilitating, and recurring effects, this vectorborne infection hobbled the effectiveness of combat forces and support staff. Various official documents and publications issued by the Office of War and by the Office of Malaria Control in War Areas, a joint undertaking by the US Public Health Service and state health departments, detailed ways to reduce malaria infection, including using antimalarial drugs, insecticides, and bed nets. But the dense, bureaucratic language in such publications did not serve as a call to action. More accessible and persuasive messaging was needed to convince military personnel to protect their health for the good of the war effort.

Complicating matters, the traditional treatment for malaria, quinine, was in short supply. In 1942, Japan had seized control of the cinchona trees grown for quinine in the Dutch East Indies and other parts of Asia, and Germany had seized control of captured quinine reserves and manufacturing facilities in Amsterdam. The Allies turned to the synthetic drug quinacrine, known as Atabrine. Although effective, Atabrine had some disagreeable side effects: it often caused diarrhea, headaches, and nausea and had the unnerving, but temporary, tendency to turn skin bright yellow. Moreover, Japanese propaganda falsely proclaimed that using Atabrine could lead to infertility. 

According to historical researcher Seth Paltzer, “It was clear to the Army that using antimalarials and insecticides were key to the fight against disease but making sure troops at the front participated in these measures continued to be a problem. As a result, a third offensive front was opened against malaria, in the form of propaganda.” Integral to that campaign were colorful, cartoonish posters for educating military personnel on malaria prevention. 

**Figure 2:**
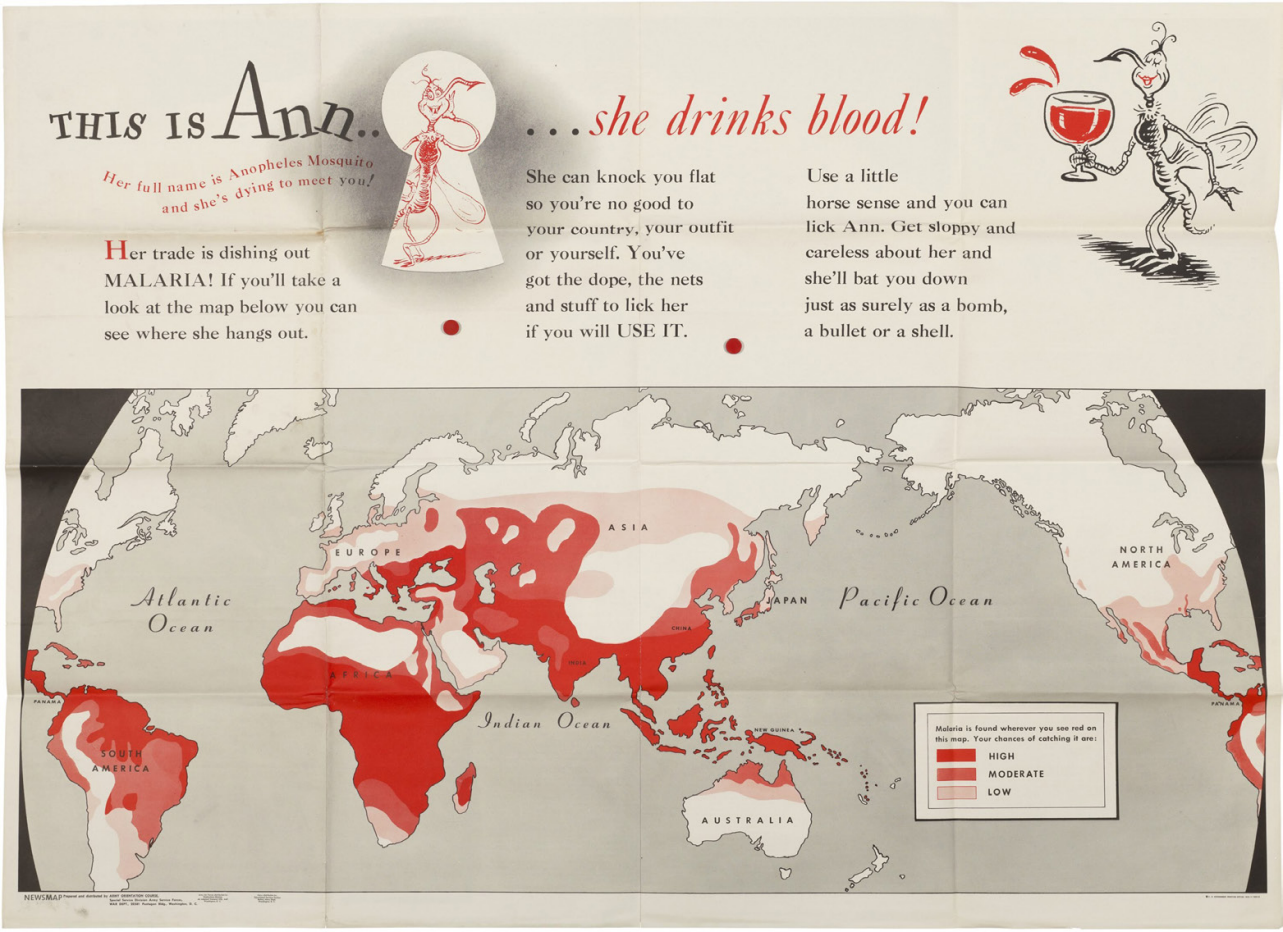
Ted Geisel (aka Dr. Seuss) illustration; Munro Leaf, text; United States War Department Special Services Division and Government Printing Office. *This is Ann–: she drinks blood!* (1943). Photomechanical print (poster): 35 inches x 47 inches/89 cm x 120 cm. National Library of Medicine, Bethesda, Maryland, United States. Public domain image.

Featured on this month’s cover is a detail of an *Anopheles* mosquito from one such poster. At the top of the poster are the eye-catching words “This is Ann . . . and she drinks blood!” Drawings of Ann, whose full name is revealed to be “Anopheles Mosquito,” appear twice, first glimpsed through a keyhole as a smiling red menace and then raising an oversized goblet brimming with blood (Figure 2). 

The informal slang-based text calls attention to a world map showing where Ann “hangs out” and warns “She can knock you flat so you’re no good to your country, your outfit or yourself. You’ve got the dope, the nets and stuff to lick her if you will USE IT.” Bands of red indicate relative risks of contracting malaria in different locations when this poster was printed in late 1943. Among the highest risk locales are the South Pacific islands and southern Italy, where American forces were deployed.

Office of War Information posters and publications do not include credits. But the cartoonish images of Ann may look familiar. They are the handiwork of the young Army Captain Theodore Geisel, best known as Dr. Seuss, the pen name he used for writing and illustrating more than 60 children’s books such as *Green Eggs and Ham* and *The Cat in the Hat*. Assigned to the Animation Department, First Motion Picture Unit, in Hollywood, California, USA, Geisel worked with a creative team of artists, cartoonists, writers, and filmmakers. Among them was Munro Leaf, another prolific author of childrens’ books, including *The Story of Ferdinand*. Leaf drafted the text for this malaria poster and collaborated with Geisel on the related booklet *This Is Ann / She’s Dying to Meet You*, featuring more of their text and illustrations. 

Ginny A. Roth, Curator of Prints & Photographs, History of Medicine Division, National Library of Medicine, notes that Geisel and Leaf believed that the various military manuals and guides explaining how to prevent malaria were “. . . boring and concluded that soldiers were either not reading them or not making a connection between malaria and mosquitoes.” 

How much difference such posters made remains speculative, but the overall campaign yielded results. Paltzer writes, “Thanks to the educational efforts of the Army’s propaganda, and the scientific and industrial base that supplied insecticides and antimalarials, the Army was able to significantly minimize the effects of malaria on the war effort, contributing in no small measure to final victory.”

Effective September 15, 1945, an executive order by President Harry Truman shuttered the Office of War Information, which he had cited for its “outstanding contribution to victory.” The war was over; however, the need to control malaria has persisted, although it has been largely controlled in many of the red-shaded areas on this WWII poster. Still the World Health Organization reports that in 2019, there were an estimated 229 million cases of malaria worldwide and an estimated 409,000 deaths, largely among children the Africa region. Ann is still drinking blood and spreading malaria, especially in resource-limited tropical and subtropical areas.
